# Biology and Treatment of Richter Transformation

**DOI:** 10.3389/fonc.2022.829983

**Published:** 2022-03-22

**Authors:** Adalgisa Condoluci, Davide Rossi

**Affiliations:** ^1^ Division of Hematology, Oncology Institute of Southern Switzerland, Ente Ospedaliero Cantonale, Bellinzona, Switzerland; ^2^ Laboratory of Experimental Hematology, Institute of Oncology Research, Bellinzona, Switzerland; ^3^ Università della Svizzera Italiana, Lugano, Switzerland

**Keywords:** Richter transformation, Richter syndrome, CLL, biology, DLBCL, Hodgkin lymphoma, treatment

## Abstract

Richter transformation (RT), defined as the development of an aggressive lymphoma on a background of chronic lymphocytic leukemia/small lymphocytic lymphoma (CLL/SLL), represents a clinical unmet need because of its dismal prognosis. An increasing body of knowledge in the field of RT is arising from the recent development of preclinical models depicting the biology underlying this aggressive disease. Consistently, new therapeutic strategies based on a genetic *rationale* are exploring actionable pathogenic pathways to improve the outcome of patients in this setting. In this review, we summarize the current understandings on RT biology and the available treatment options.

## Definition of Richter Transformation

Richter transformation (RT) is defined as the development of a high-grade lymphoma in patients with a previous or concurrent diagnosis of chronic lymphocytic leukemia/small lymphocytic lymphoma (CLL/SLL) (1).

RT was originally depicted as a ‘reticular cell sarcoma’ with presence of ‘leukemic and tumor cells’ on a lymph node biopsy from a male patient with CLL and rapid clinical deterioration by Maurice N. Richter in 1928 (2). The occurrence of secondary aggressive lymphomas on a CLL background took the definition of ‘Richter transformation’ in 1964, when a case series of 14 patients with CLL developing malignant reticulopathy was described by Lortholary and colleagues (3).

The estimated incidence of RT in patients with CLL/SLL previously treated with chemo/chemoimmunotherapy was reported to be 0.5–1% per year (4). Different histopathologic variants of RT have been described in the literature, ranging from the more common diffuse large B-cell lymphoma subtype (DLBCL-RT) which accounts for up to 90–95% of RT cases, to the less represented Hodgkin lymphoma subtype (HL-RT) accounting for up to 5–10% of cases (1). Few cases (<1%) of plasmablastic transformation have been also reported (5).

## Epidemiology and Clinical Features

The large variability of the reported prevalence of RT (1–23%) has been related to different factors, mainly depending on the diagnostic assessment of RT (biopsy-proven or just clinically suspected), and on the setting (clinical trials involving fit patients or real-world data) from which data were derived (5–8).

Recently, the German CLL Study Group (GCLLSG) reported a 3% prevalence of RT in a cohort of 2,975 patients with CLL longitudinally monitored after their enrolment in clinical trials (9).

Data coming from the SEER database on 74,116 patients with CLL diagnosed between 2000 and 2016, depicts a 0.7% incidence of transformation, mostly emerging with nodal involvement (74%) (10). The gastrointestinal tract, the skeletal system, and the brain/CNS are the most commonly reported extra-nodal sites, being described in 25, 19, and 12% of cases, respectively. Median time to transformation is 1.8–1.9 years for DLBCL-RT (3, 11) and 4.6–7.5 years for HL-RT (12, 13), even if no significant difference according to different histotypes is reported in other datasets (10).

A higher incidence of RT has been reported for highly pretreated relapsed/refractory (R/R) CLL patients enrolled in the first clinical trials with novel agents (2–15%), while in first-line the incidence of RT is 0–4% in this treatment setting (13–21). However, these data refer to short follow-up periods and longer observation time is needed to properly evaluate the impact of chemo-free treatments on second malignancies/transformation. Similar clonal evolution patterns are described for patients experiencing transformation under novel agents or chemo-immunotherapy (CIT) (22, 23).

## Diagnosis

Rapid physical deterioration and/or occurrence of B symptoms (i.e., fever with no infectious background, weight loss), rapid and localized growth of lymph nodes, rise in lactate dehydrogenase (LDH) levels, and hypercalcemia, are all signs that should raise suspicion for aggressive transformation, particularly in a patient with known CLL. However, these clinical findings are specific for RT in only 50–60% of cases, the remaining ones being manifestations of histologically aggressive CLL (aCLL) or solid cancers (24).

The gold standard for RT diagnosis is histologic documentation with an open biopsy. Fine needle biopsy may not illustrate the whole lymph node structure, leading to false positive diagnoses (i.e., expanded proliferation centers may be seen in fine needle biopsies from patients with progressive or aCLL) (25).

### Role of ^18^FDG PET/CT

Since RT is often limited to one single lesion at the time of evolution, any biopsy aimed at confirming RT should be directed at the ‘index’ lesion (the lesion showing the most active dimensional dynamics). ^18^FDG PET/CT may assist in the choice of whether and where to perform a biopsy (24, 26, 27). When a standard uptake value (SUV) cut-off of 5 is chosen, the high negative predictive value (97%) of the ^18^FDG PET/CT in this setting supports a non-biopsy approach for lesions with SUV <5. Given the limited positive predictive value (53%) of ^18^FDG PET/CT for lesions with an SUV ≥5, the biopsy should be performed at the site of the index lesion (24, 26, 27).

A higher positive predictive value (60.6%) has been described when establishing an SUV cut-off of 10, with a sustained elevated negative predictive value (99.2%) and a good correlation with overall survival (OS). Patients with lesions displaying an SUV ≥10 showed a median OS of 6.9 months, while for patients displaying lesions with an SUV <10 the reported median OS was 56.9 months (28). However, for patients with RT arising after kinase inhibitor therapy, the SUV threshold of 10 showed lower negative predictive values (50%) (29).

### Morphology and Immunophenotype

#### Morphology of RT Subtypes

The presence of confluent sheets of large neoplastic B lymphocytes characterizes the morphology of the DLBCL-RT (4, 30). Notably, an enlargement of proliferation centers in lymph nodes can occur also in the ‘aggressive’ or ‘accelerated’ CLL (aCLL), which needs to be distinguished from the proper transformation, as it is associated with an outcome intermediate between typical CLL and classic RT (4). Morphologic discrimination of RT from aCLL is mainly based on the characteristics of B-cells nuclei and growth pattern (a nuclear size equal or larger than macrophage nuclei or >2× a normal lymphocyte and a diffuse growth pattern are more typical for RT) (31, 32).

The HL-RT subtype is characterized by the presence of Reed–Sternberg cells either in a typical background of small T cells, epithelioid histiocytes, eosinophils and plasma cells or scattered in a background of CLL cells (4, 30, 33).

#### Phenotype

DLBCL-RT cells express CD20, and less typically CD5 (~30% of cases), or CD23 (~15% of cases) (4, 34). PD-1 expression is described in DLBCL-RT neoplastic B-cells, while a weak expression is restricted on the paraimmunoblasts of proliferation centers of CLL samples and rarely found in *de novo* DLBCL specimens (35, 36). The positivity of transformed B-cells for PD-1 showed a 90% correlation with molecularly defined clonal relationship between CLL and DLBCL-RT. Accordingly, PD-1 expression has been proposed as a candidate surrogate for defining the clonal relationship of DLBCL-RT (35).

#### HL Variant

Hodgkin and Reed–Sternberg cells show a characteristic CD30^+^/CD15^+^/CD20^−^ immunophenotype and are often EBV positive (4, 34).

### Clonal Relationship Between RT and the Underlying CLL

The definition of clonal relationship between RT and the underlying CLL relies on the analysis of the rearrangement of IGHV-D-J genes [by PCR or next-generation sequencing (NGS) methods]. Most cases of DLBCL-RT (~80%) are clonally related to the previous CLL phase, representing true transformations (34, 37). Clonally unrelated cases represent *de novo* DLBCL arising in a patient with concomitant CLL, and are usually described on an IGHV-mutated CLL background (4). Clonal relationship impacts meaningfully on the prognosis of patients with DLBCL-RT, with clonally related cases showing a median OS of less than 1 year. Conversely, for patients with clonally unrelated RT the reported survival is ~ 65 months, similarly to *de novo* DLBCLs (6, 30).

Clonal relationship between HL-RT and the underlying CLL has been reported in only 30% of cases (30).

## Biology of RT

Genetic alterations leading to RT are progressively being described for DLBCL-RT, which displays some common characteristics with other transformed lymphomas. Less is reported on HL-RT, whose molecular background and behavior are similar to *de novo* HL.

### Biology of DLBCL-RT

Somatic alterations involving genes of tumor suppression, cell cycle and proliferation pathways (i.e., mutations or disruptions of *TP53*, *NOTCH1*, *MYC*, and *CDKN2A*) are the main genetic clues of DLBCL-RT and can explain its aggressive disease kinetics and chemoresistance (30, 37, 38).

*TP53* is a master regulator of the DNA-damage-response pathway, and leads to cell apoptosis if activated (i.e., as in response to the antiproliferative effect of chemotherapies). *TP53* mutations/deletions can be acquired at the time of transformation and are the most frequent genetic lesions of DLBCL-RT, being described in 60% cases (38).

*MYC* is involved in a transcription regulating network and is found altered in ~40% of DLBCL-RT (11, 30, 37–39).

*CDKN2A* is a negative regulator of cell cycle transition from G1 phase to S phase and can be deleted in 30% of RT cases (30, 38). The rapid kinetics and aggressive behaviour of RT may be explained by cell cycle deregulation linked to *CDKN2A* alterations. It has been recently demonstrated that a concomitant loss of function of *TP53* and *CDKN2A*/*CDKN2B* enables a B-cell receptor (BCR)-dependent proliferation of large pleomorphic cells with a diffuse RT-like morphology (40).

The biased usage of subset 8 configuration in the BCR has been associated to *NOTCH1* somatic mutations. This molecular setting allows for autonomous BCR signaling and a dynamic responsiveness of neoplastic B cells to auto-antigens and/or immune stimuli from the microenvironment (33, 41). The reported 5-year rate of transformation for patients with CLL and subset 8 usage is ~ 70% (31).

*NOTCH1* mutations represent the only validated risk factor for RT. The reported cumulative risk of developing DLBCL-RT is 45% among patients with CLL and mutated *NOTCH1*, while it is 4% for CLL with wild-type *NOTCH1* (42–44).

Mutational whole-genome sequencing (WGS) data from paired circulating CLL and RT biopsies were reported and independently confirmed by RNA expression profiling for 17 patients diagnosed with DLBCL-RT. RT was characterized by mutations in the DNA damage pathway and in poor-risk CLL drivers (45). *TRAF3* (a signaling regulator), *NF-kB*, and mitogen-activated protein kinase pathways, were reported to commonly harbor heterozygous deletions (45). *PTPN11*, a positive regulator of the MAPK–RAS–ERK signaling pathway, was overexpressed in RT samples (45). *SETD2* (showing alterations in ~30% of RT samples) and *PTPRD*, a tumor suppressor gene found silenced in many cancers *via* hypermethylation, were recurrently deregulated. Compared with the paired CLL, RT samples were characterized by increased mutational burden mainly related to some genes previously unrecorded in CLL (*BDKRB1*, *WWP1*, *TFCP2*, *SVIL*, *SLC9B1*, *RELN*, *PTK2*, *IRF2BP2*, *IL7*) (45), and whose role in RT pathogenesis needs to be clarified by functional studies. Further mutations were described in non-coding regions of immune-regulatory genes (i.e., *BTG2*, *CXCR4*, *NFATC1*, *PAX5*, *NOTCH1*, *SLC44A5*, *FCRL3*, *SELL*, *TNIP2*, and *TRIM13*), suggesting their potential role in RT pathogenesis (45). Consistently, distinct immune signatures between peripheral blood and lymph nodes from patients with RT have been depicted in another study (46). A low T-cell TCR clonality was found in peripheral blood, with a consequent high diversity of the T cell repertoire and a potentially active host immune response. RT samples were characterized by enhanced PD-L1 expression in histiocytes and PD-1 in neoplastic B cells, and also infiltration of FOXP3-positive T cells and CD163-positive macrophages. These findings depict a peculiar RT-immune microenvironment and may explain the higher response rates to immune checkpoint inhibitors (47).

According to the model proposed by Teng et al. to classify tumor microenvironments based on PD-L1 expression in tumor cells and tumor-infiltrating lymphocytes (TIL), RT may harbor a type I microenvironment (PDL1^+^, TIL^+^), reflecting an adaptive immune resistance environment, which can be the target of checkpoint inhibitors (48, 49). CLL, on the other hand, seems to be characterized by immunological ignorance defined as type II microenvironment (PD-L1^−^, TIL^−^) with poor expected response from checkpoint suppressors (47–49).

An increased *LAG3* gene expression has been reported in RT, with respect to *de novo* DLBCL and other transformed lymphomas (50). LAG3 membrane protein is expressed on both neoplastic B cells and/or TILs and is involved in the delivery of inhibitory stimuli on activated T cells. In RT, *LAG3* shows a strong positive correlation with HLA Class II and immune regulatory genes (namely, *TIGIT* and *PD-1*), with an immune microenvironment characterized by potential adaptive immune resistance when *LAG3* is overexpressed (51, 52).

Constitutive phosphorylation of AKT is higher among patients with CLL at high risk for RT transformation (i.e., CLL with *NOTCH1* mutation, aggressive CLL with *TP53* disruption) (53). In a new experimental TCL1 mouse model of CLL with a constitutively active Akt allele (Akt-C) in B cells, the development of an aggressive lymphoma and a massive splenomegaly was reported by the age of 7 months confirming the driving role of *AKT* for RT-like transformations. Akt-C mice showed a highly expressed NOTCH signaling, with an expansion of CD4 T cells expressing DLL1 (the NOTCH1 ligand present on T cells) in the microenvironment. This upregulation has been related to the *NOTCH1* activation of tumor cells, accordingly to their commitment for transformation.

Regulating the homing of immune cells, the CXCR4–CXCL12 axis is crucial for the interaction of CLL cells and microenvironment (54–57). In the Eμ-TCL1 mouse model, the introduction of a gain-of-function *Cxcr4* mutation (*Cxcr4* ^C1013G^) that hyperactivates CXCR4 signaling, led to cell cycle dysregulation *via PLK1*/*FOXM1* (58). These neoplastic cells showed a transcriptional signature similar to that of patients with RT.

**Figure 1:**
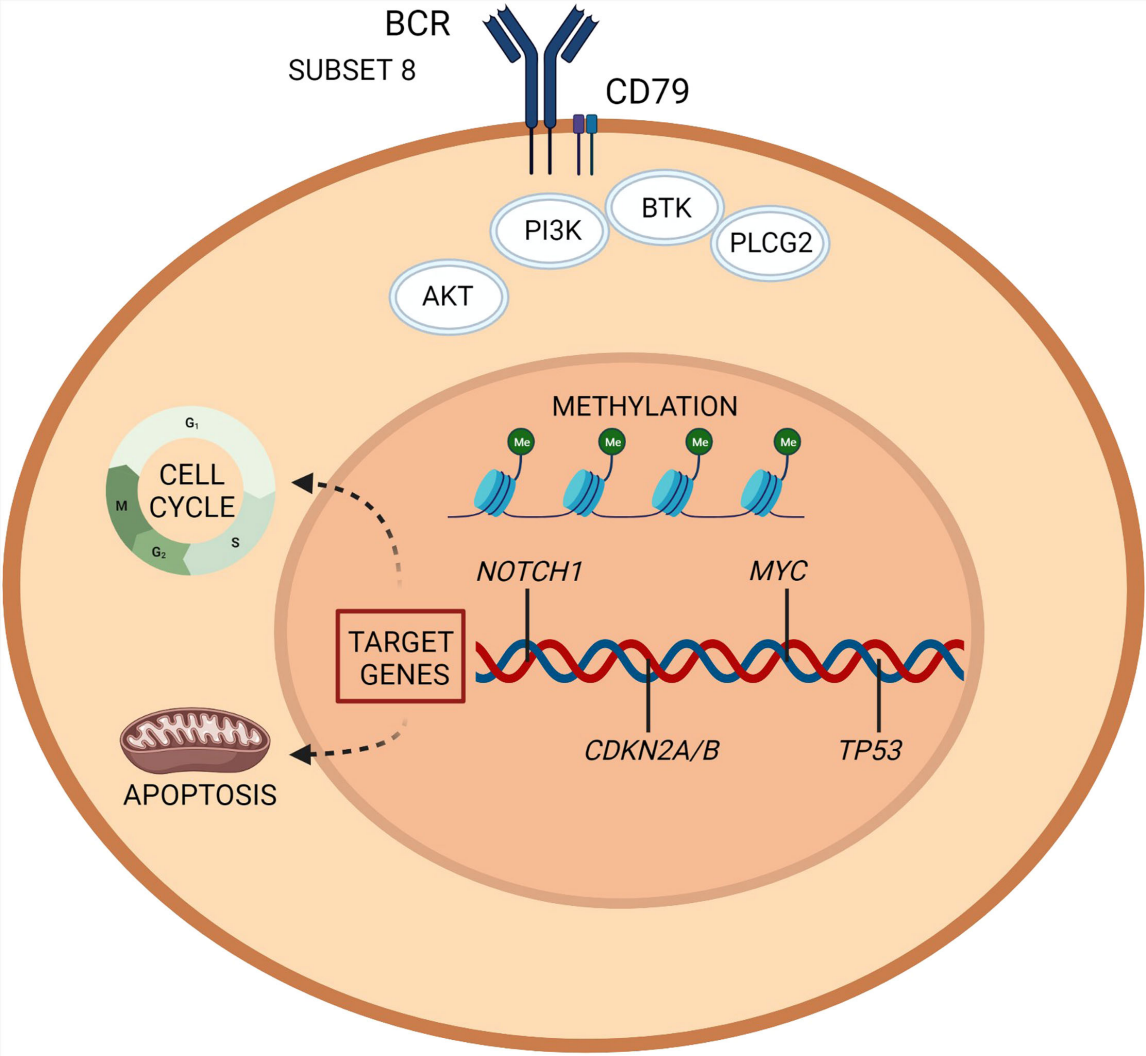
Richter transformation: intrinsic vulnerabilities and targets for treatment. A representation of the molecular pathogenesis of Richter transformation, resulting from a number of epigenetic and genetic lesions occurring in the tumor cell population. Recurrently mutated genes affect DNA repair, B cell receptor, and chromatine modification. Created with BioRender.com.

**Figure 2:**
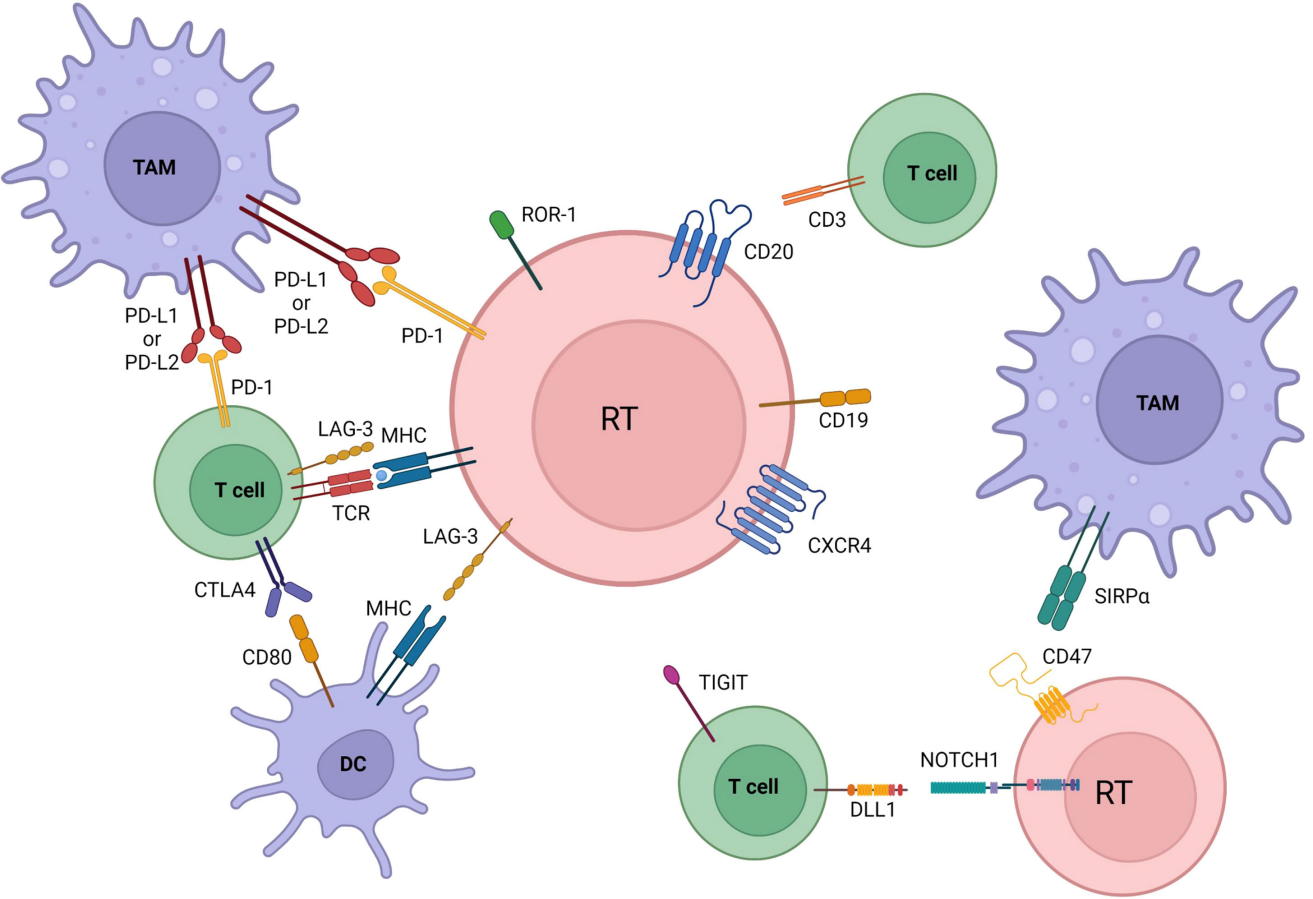
Microenvironmental crosstalks and druggable targets in Richter transformation. Pathway activation and changes in immune checkpoints profile are also involved in transformation. Communication between the tumoral cells, dendritic cells, tumor associated macrophages (TAM), and T cells is established by direct contact, chemokine/cytokine receptors, adhesion molecules and ligand-receptor interactions. Immune inhibitory molecules (PD-L1 among others) facilitate tumor cells to evade immune-response and maintain tolerance. All of the here represented are druggable targets in RT. BCR, B cell receptor; DC, dendritic cells; TAM, tumor associate macrophage. Created with BioRender.com.

The main pathways with a reported involvement in RT pathogenesis are resumed in **Table 1** and **Figures 1**, **2**.

**Table 1:** Summary of the main biomarkers involved in DLBCL-RT pathogenesis.

Biomarker(s)	Frequency	Role	Consequence	Note	Reference
*Biased usage of BCR subset 8*	8%	BCR signaling	Autonomous signaling and increased response to auto-antigens and immune stimuli	5-years transformation rate of patients with CLL and subset 8 usage: ~70%	(33, 41)
*TP53*	60%	Regulation of DNA-damage-response pathway	inactivation	Impaired apoptosis in response to the antiproliferative effect of chemotherapies due to *TP53* loss may explain the chemorefratoriness of RT	(38)
*MYC*	40%	Regulation of transcription network	Overexpression	Key transcription factor which regulates up to 15% of human genes, constantly involved in transformation from indolent to aggressive lymphomas	(11, 30, 37–39)
*CDKN2A*	30%	Regulation of cell cycle	Inactivation	Concomitant loss of function of *TP53* and *CDKN2A/B* leads to BCR-dependent proliferation of abnormal B cells	
*NOTCH1*	40%	NFκB activation	Activation	*NOTCH1* gene have been reported in ~10% of patients with CLL at diagnosis, mainly those with CLL of the IGHV-UM	(42–44)
*AKT signaling*	>50%	Driver of protein synthesis, cell survival, proliferation, and glucose metabolism	Activation, constitutive phosphorylation	AKT is activated in high-risk CLL and in >50% of patients with RT. Constitutive AKT may amplify the NOTCH1 signal or add additional signals that accelerate transformation	(53)
*SETD2*	30%	Histone methyltransferase that catalyses the trimethylation of lysine 36 on histone 3 (H3K36me3), epigenetic regulator of gene transcription	Inactivation	Deletions and mutations in ~7% of CLL patients requiring treatment	(45)
*TRAF3*	–	Signaling regulator, namely, Toll-like receptor signaling, NF-κB, and mitogen-activated protein kinase pathways	Inactivation	TRAF3 deficiency enhances survival of B cells and increases transformation risk *via* upregulation of PIM3 and c-MYC expression	(45)
*PTPN11*	–	Regulator of MAPK-RAS-ERK pathway	Activation	Rare CLL driver	(45)
*PTPRD*	–	Tumor suppressor colocated with *CDK2NA*	Inactivation	Receptor protein tyrosine phosphatase regulating cell growth	(45)
*LAG3*	–	Membrane protein expressed in B cells and/or TILs	Increased gene expression	Immune checkpoint gene. LAG3 protein is expressed on immune cells and in the setting of persistent antigen exposure; co-expressed with other immune checkpoints in dysfunctional T cells.	(50)
*CXCR4*	–	G-protein-coupled receptor regulating hematopoietic stem cell homeostasis, myelopoiesis, lymphopoiesis, and homing of immune cells toward its ligand C-X-C motif chemokine 12 (CXCL12)	Activation *via PLK1*/*FOXM1*	Involved in the migration and trafficking of malignant B cells	(58)

BCR, B-cell receptor; CLL, chronic lymphocytic leukemia; TIL, tumor infiltrating lymphocytes; RT, Richter transformation.

## Prognosis of RT

The DLBCL-RT prognosis is overall poor, with a reported median OS of 10 months (10). As already described, the most impactful prognostic factor is the clonal relationship between the transformed DLBCL and the underlying CLL (see section *Clonal Relationship Between RT and the Underlying CLL*).

### Prognostic Scores

The RT prognostic score based on clinical and laboratory variables (Zubrod performance status >1, increased LDH levels, platelets ≤100× 10^9^/L, tumor size ≥5 cm, and >2 prior lines of therapy) allows to differentiate 4 risk groups, with a median survival of 13–45 months for low risk patients (0–1 risk factors); 11–32 months for low-intermediate risk (2 risk factors); 4 months for high-intermediate risk (3 risk factors); 1–4 months for high risk patients (4–5 risk factors) (59).

Complex karyotype (CK) diagnosed on the underlying CLL has a negative impact on RT-related outcome (60). Type-2 CK (CK2, CK with major structural abnormalities) or high-CK (CK with >5 chromosome abnormalities), together with IGHV unmutated status, 11q deletion, *TP53* disruption and Binet stage B/C, have been identified as predictors for RT prognosis. According to the Richter syndrome scoring system, patients with high-CK and/or CK2 show a 10-year risk of developing RT of 31%; patients with unmutated IGHV/11q deletion/*TP53* disruption/>B Binet stage show a 10-year risk of 12%; while patients with mutated IGHV without CK and with wild type *TP53* display a 10-year risk of developing RT of only 3% (60).

### Role of Previous Treatment

Longer survival is reported for patients with treatment-naïve CLL when compared to the relapsed/refractory setting (12 vs 7 months) (10, 61–65). RT after ibrutinib or venetoclax shows an aggressive behavior. The median OS after progression for double class-resistant CLL patients (i.e., CLL resistant to both BTK and BCL2 inhibitors) is 3.6 months, and this class of patients represents a clinical unmet challenge in the era of novel agents (66).

## Treatment of DLBCL-RT

**Figure 3:**
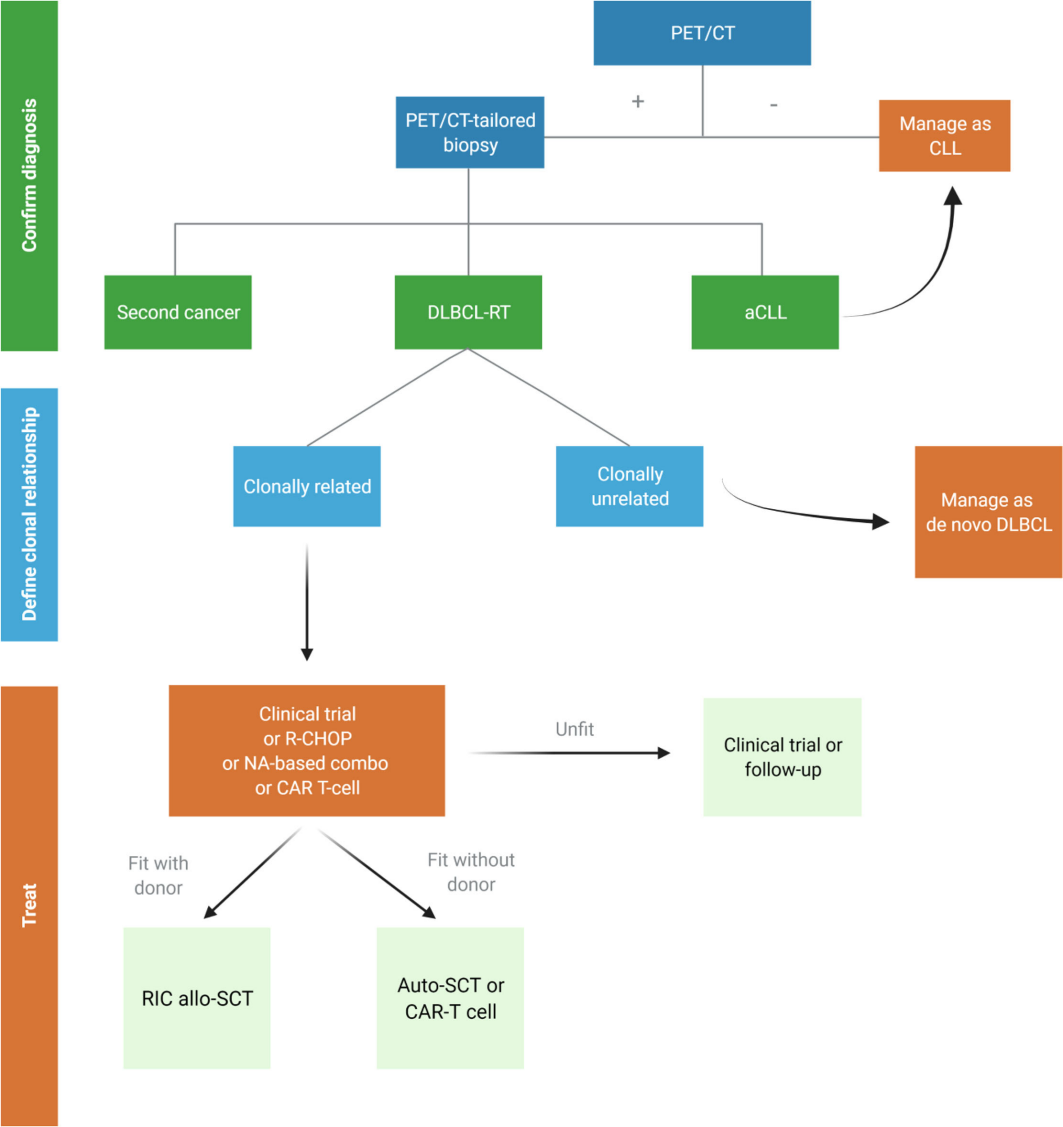
Proposed algorithm for the management of suspected diffuse large B-cell Richter transformation (DLBCL-RT). aCLL, accelerated chronic lymphocytic leukemia; auto-SCT, autologous stem cell transplantation; DLBCL, diffuse large B-cell lymphoma; NA, novel agents; RIC allo-SCT, reduced intensity conditioning stem cell transplantation. Created with BioRender.com.

History and comorbidities of patients developing RT drive the choice of treatment in this challenging setting. A proposed algorithm for DLBCL-RT is depicted in **Figure 3**.

### Chemo-Immunotherapy

Translating treatment experience from the aggressive B-cell non-Hodgkin lymphoma setting, combinations of anti-CD20 monoclonal antibodies and polychemotherapy regimens have been indicated to treat patients with DLBCL-RT.

The historical standard regimen for DLBCL R-CHOP (rituximab, cyclophosphamide, doxorubicin, vincristine and prednisone) produced response rates of up to 67% (complete response, CR 7%), reaching a median progression free survival (PFS) of 10 months and a median OS of 21 months. Reported adverse events were mainly hematological (65%), while severe infections were described in 28% of patients (67). Another case series reports data on 48 patients with DLBCL-RT treated with R-CHOP with a response rate of 37% and a median OS of 35 months (9).

The combination of CHOP chemotherapy with the anti-CD20 ofatumumab (O) showed an overall response rate (ORR) of 46% (CR 27%), a median PFS of 6 months and a median OS of 11 months. Reported adverse events were infections and hematologic toxicities (thrombocytopenia, febrile neutropenia, sepsis) (68, 69).

More aggressive CIT regimens were assessed, though not achieving an improved outcome. R-EPOCH (rituximab, etoposide, prednisone, vincristine, cyclophosphamide, and doxorubicin), a regimen indicated in high grade B-cell lymphoma, reached a response rate of only 20%, a median PFS of 3 months and a median OS of 6 months (70). Shorter PFS and OS were observed in patients with disrupted *TP53* and an underlying CLL characterized by complex karyotype.

Poor median OS and response rates of 40% were reported with the hyper-CVAD regimen (fractioned cyclophosphamide, vincristine, doxorubicin, and dexamethasone), alone or in alternating combination with methotrexate and ara-C. Severe hematotoxicity, high infection rates (developed by 50% of patients) and a treatment-related mortality of nearly 20% were reported (71), even under the proper prophylaxis with granulocyte–macrophage colony stimulating factor (GM-CSF) (72).

The OFAR 1 and 2 trials explored the combination of oxaliplatin, fluradabine, ara-C and rituximab at different dosages to prevent toxicities. The ORR ranged from 39 to 50%, being characterized by a median PFS of 3 months and a median OS of 6–8 months (73, 74). The main complication was myelotoxicity, with no significant improvement in myelosuppression severity for patients enrolled in the OFAR 2 trial compared to the OFAR 1 trial (74).

### Consolidation With Stem Cell Transplantation

Due to the high rate of relapses and poor OS after CIT, stem cell transplantation (SCT) has been proposed as a consolidation strategy for DLBCL-RT. The benefit of receiving SCT is underlined by a median survival of 5 years vs <1 year for patients not undergoing SCT, and relies on high-dose cytotoxic therapy combined to a graft-versus-leukemia effect in the case of allogeneic SCT. The latter is confirmed by a plateau in relapse-free survival curves after allogeneic SCT (75).

However, most patients (~85%) cannot access SCT, either due to their poor performance status, a refractory disease to induction treatments, and/or the lack of donor availability (75).

The Center for International Blood and Transplant Research (CIBMTR) registry study evaluated outcomes in 53 and 118 patients with DLBCL-RT treated with autologous SCT and allogeneic SCT, respectively. A 37% relapse incidence, 48% PFS, and 57% OS at 3 years was reported in the autologous SCT cohort. For patients treated with allogeneic SCT, relapse incidence, PFS, and OS at 3 years were 30, 43, and 52%, respectively. In the latter cohort, outcomes strongly correlated with the response status at SCT (3-year OS 77% for patients reaching a CR with induction therapy versus 57% for partial responses), while cytogenetic abnormalities and prior novel therapy did not show an impact on survival (76).

A single-center retrospective analysis of 23 RT patients undergoing reduced intensity conditioning (RIC-SCT) reports a 5-year PFS of 40% and OS of 58% (77). Young age (<60 years), deeper response at SCT and having received <3 previous lines of therapy positively correlated with outcomes, while cytogenetic/molecular features and exposure to novel agents did not show an impact on PFS/OS (77, 78). Total body irradiation (TBI) resulted in poorer outcomes (77).

A median OS of 17 months has been recently reported by GCLLSG for 3 patients undergoing allogeneic SCT for RT (9).

In a meta-analysis evaluating the outcome of patients with RT undergoing allogeneic SCT, the relapse rate was 28% and the non-relapse mortality 24%, showing similar rates previously reported for patients diagnosed with other lymphoproliferative diseases (78).

Overall, young and fit patients with DLBCL-RT attaining deep responses with induction treatment can benefit both from autologous SCT and RIC allogeneic SCT, while TBI-containing RIC should be considered with caution.

### Novel Agents

Recent advances in the understanding of deregulated molecular pathways in RT led to investigate the efficacy of targeted agents, with promising results.

XPO1 is a nucleo-cytoplasmic transporter of tumor suppressor proteins, whose activity is often upregulated in cancers. Selinexor, a selective inhibitor of nuclear export, acts with the aim of maintaining tumor suppressors within the nucleus to preserve their activity. In DLBCL-RT selinexor produced a response rate of 33% with an acceptable toxicity profile (79). Unfortunately, the phase 2 study (NCT02138786) was closed prematurely due to enrolment hurdles.

Bruton’s tyrosine kinase (BTK), a component of BCR, plays a central role in B-cell malignancies, regulating cell proliferation and survival. Ibrutinib, the first-in-class BTK inhibitor, showed activity in DLBCL-RT (80–82), with a survival benefit and a 16 months PFS (82). Responses to ibrutinib rechallenge have been reported after incidental RT diagnosis upon ibrutinib discontinuation in three patients with CLL (83). Acalabrutinib is a second generation oral BTK-inhibitor with an ORR of 40% (CR 8%) (84) and a median PFS of 3 months. The phase 1/2 BRUIN study (NCT03740529) evaluated safety and efficacy of pirtobrutinib (loxo-305), a next generation, highly selective, non-covalent BTK inhibitor in previously treated RT (85). Among 15 patients, pirtobrutinib reached a response rate of 67% (CR 13%). The 6-month PFS rate was estimated to be 52%. The median number of prior lines of system therapy was 6, with 82% of DLBCL-RT patients having received a prior BTK inhibitor, 59% a prior BCL-2 inhibitor, and 6% CAR T-cell therapy.

The reversible BTK inhibitor nemtabrutinib (previously known as ARQ531 or MK-1026) showed efficacy in *in vivo* BTK-resistant CLL/RT models (i.e., Eμ-MYC/TCL1 murine model recapitulating the disease phenotype of RT) (86, 87). Inhibitory activity of ARQ531 on the BCR pathway was reported both upstream and downstream of BTK *via* SYK, AKT, and MEK1/ERK. This effect was maintained also in presence of the C481S *BTK* resistance mutation and autoactivating *PLCγ2* mutations. Safety and activity profile of nemtabrutinib are being explored in ongoing clinical trials enrolling patients with B-cell malignancies, including RT (NCT03162536, NCT04728893) (see **Table 2**).

**Table 2:** Ongoing trials with targeted agents in diffuse large B-cell Richter transformation.

Interventions	Targeted pathway and/OR Antigen	Ref.
Acalabrutinib + R-CHOP	BTK	NCT03899337
Ibrutinib + DA-EPOCH-R	BTK	NCT04992377
Venetoclax + DA-EPOCH-R	BCL-2	NCT03054896
Blinatumomab after R-CHOP	CD19	NCT03931642
Polatuzumab vedotin + DA-EPOCH-R	CD79b	NCT04679012
Epcoritamab	CD3/CD20	NCT04623541
Nemtabrutinib (ARQ 531)	BTK	NCT03162536
		NCT04728893
Ibrutinib + Nivolumab	BTK + PD-1	NCT02420912
Zanubrutinib + Tislelizumab	BTK + PD-1	NCT04271956
Duvelisib + Nivolumab	PI3K + PD-1	NCT03892044
Copanlisib + Nivolumab	PI3K + PD-1	NCT03884998
Duvelisib + Venetoclax	PI3K + BCL-2	NCT03534323
Umbralisib + Ublituximab	PI3K, CK1 + CD20	NCT02535286
Obinutuzumab + Ibrutinib + Venetoclax	CD20 + BTK + BCL-2	NCT04939363
Atezolizumab + Obinutuzumab + Venetoclax	PD-L1 + CD20 + BCL-2	NCT02846623
Atezolizumab + Obinutuzumab + Venetoclax	PD-L1 + CD20 + BCL-2	NCT04082897
Ipilimumab + Ibrutinib + Nivolumab	CTLA-4 + BTK + PD-1	NCT04781855
TG-1801 + Ublituximab	CD47/CD19 + CD20	NCT04806035
ALX148 + Rituximab + Lenalidomide	CD47 + CD20	NCT05025800
VIP152	CDK9	NCT04978779
Zilovertamab vedotin (VLS101)	ROR1	NCT03833180
CD19 CAR-T cell	CD19	NCT04892277
CD19 CAR and PD-1 Knockout T Cells	CD19	NCT03298828
CAR70/IL15 NK cells	CD70	NCT05092451

Patients with *TP53*/*NOTCH1*-disrupted high-risk CLL and RT display increased constitutive AKT phosphorylation (88). Some activity data has been reported with the PI3K inhibitor idelalisib in patients with RT (89), prompting further investigation of these agents in this condition.

Considering that DLBCL-RT harbors *TP53* alterations, novel treatments and combinations in this setting need to act in a *TP53*-independent way. Venetoclax inhibits BCL2 and is strongly active in high-risk CLL, acting independently from *TP53* (90). In the M12-175 (NCT01328626) phase I study, 7 patients with DLBCL-RT were treated with escalating doses of venetoclax, attaining a response rate of 43% (no CRs reported) (90). In the phase 2 study on the combination venetoclax-R-EPOCH (NCT03054896), the ORR reached 62% (42% CR with unmeasurable residual CLL in bone marrow). Median PFS and median OS were 10.1 and 19.6 months, respectively. Main adverse events were related to grade 3–4 neutropenia (65%), thrombocytopenia (50%) and febrile neutropenia (38%). No tumor lysis syndrome (TLS) occurred with daily venetoclax ramp‐up after 1 lead in cycle of R‐EPOCH (91).

Immune checkpoint deregulation is common in the setting of DLBCL-RT, which frequently develops upon an exhausted immune system. Immune checkpoint blockade with the monoclonal anti-PD1 antibody pembrolizumab produced 44% response rate (NCT02332980) (47). Importantly, responses were observed only in patients previously exposed to ibrutinib, with a median OS not reached (median OS of 10.7 months for the whole cohort). Preclinical studies reported synergistic antitumor effects between BTK and the PD-1/PD-L1 inhibitors (92). Ibrutinib exerts immune modulating effects through IL-2 inhibition, deregulating T-cell proliferation and differentiation signaling. The combination of ibrutinib with nivolumab (an anti-PD1 antibody) was assessed in patients with relapsed or refractory hematological malignancies, namely, high-risk CLL/SLL, follicular lymphoma, DLBCL, and RT (93). The ORR was 65% in the DLBCL-RT cohort (10% CR), with a median duration of response of 6.9 months. A phase 2 trial is exploring the combination of the anti-PD-L1 antibody atezolizumab with venetoclax and the anti-CD20 antibody obinutuzumab in patients with untreated or R/R RT (NCT02846623). Venetoclax treatment is introduced at cycle 2, after obinutuzumab + atezolizumab lead-in. Data from this ongoing trial report an ORR of 100% (71% CR) for the first 7 patients with untreated RT enrolled, with responses achieved early after the introduction of venetoclax (94). After a median follow-up of 11.2 months, three of the complete responders underwent consolidation with allogeneic-SCT and no fatalities were reported.

Glofitamab is a T-cell-engaging bispecific antibody with a 2:1 anti-CD20/CD3 structure, that has been investigated in a phase I study enrolling patients with R/R non-Hodgkin lymphoma (*de novo* DLBCL, transformed follicular lymphoma, primary mediastinal B-cell lymphoma, mantle cell lymphoma, and RT). In this study, the reported ORR and CR rates were 48 and 33%, including 41 and 28% in patients with DLBCL (95). Cytokine release syndrome (CRS) was the most common adverse event (25% grade 3, 2% grade 4), and its incidence increased with higher doses but declined after the first administration (13% events at cycle 2, 6% at cycle 3 or later).

CD19 is a transmembrane protein found invariably on B cells (except for plasma cells) with a pivotal role in BCR signaling (96). Its sustained expression even upon tumoral transformation of B cells led to the development of CAR T-cell targeting its surface antigenic domain (97, 98). It should be noted that a proportion of patients relapsing after treatment with CD19 CAR-T cells may develop a CD19^-^/CD19^dim^ disease as a mechanism of escape (99–101). In the setting of DLBCL-RT, CD19 CAR-T cells showed response rates at 4 weeks after infusion ranging from 71 to 83% (101–103) and a 1-year OS and PFS of 86 and 59%, respectively (102). In one of these studies 8 patients with RT after chemoimmunotherapy and therapy with BTK and/or BCL2 inhibitors were enrolled (103). Patients received locally produced 1 × 10^6^ autologous CD19 CART-cells/kg, modified with retroviral vector encoding a CAR comprising FMC63 anti-CD19 ScFv linked to a CD28 costimulatory domain, and CD3-zeta intracellular signaling domain. RT patients receiving CD19-CAR T-cells had a median age of 64 years (median age at CLL diagnosis 56 years), being previously treated with a median of 3 lines of therapy for CLL. On day 28 a complete response was reported in all the responders (71%, 5/8 patients). After median follow up of 6 months, two patients proceeded to allogeneic-SCT. CRS grade ≥3 requiring tocilizumab was described in 3/8 patients, while grade 3 central nervous system (CNS) toxicity was experienced by two patients.

Higher response rates (8/9 DLBCL-RT patients) are reported using axicabtagene ciloleucel CAR-T cell therapy (104). Of these patients, 8 were previously treated with kinase inhibitors and one patient died due to an infection. A CR was reported for 5/8 patients, while a partial response was described in 3 patients.

In another phase 1 study, four patients with RT were treated with escalating doses of autologous 19-28z/4-1BBL+ CAR T cells (NCT03085173) (105). Of the responders, 2/3 achieved CR and no severe CRS was reported.

ARI-0001 are autologous T-cells transduced with a CD137-based CAR construct targeting CD19 and developed at the Hospital Clinic of Barcelona (106). The CAR-T product ARI-0001 was administered to six patients with RT (five patients with DLBCL-RT and one patient diagnosed with plasmablastic transformation), achieving CRs in three patients sustained at 1.4, 12.5, and 26.7 months after treatment, respectively. With a median follow-up of 5.6 months, one patient had a stable disease, and two patients experienced a CD19-negative relapse despite no prior anti-CD19 therapy. The safety profiled was considered acceptable, with only one fatality reported due to the COVID pandemic in a patient not being treated.

Natural killer (NK) cells belong to the innate immune system and play a central role in immune surveillance. Their manageability relies upon the possibility to administer them without the need for full HLA matching, even when obtained from an allogeneic source (i.e., cord blood) (107). In the setting of CAR-engineering, this translates into an easier manufacture since there is no need to generate a patient-specific product. CAR-NK cells derived from cord blood and transduced with anti-CD19 CAR, interleukin-15, and inducible caspase 9 were explored in patients with CD19^+^ lymphoid tumors including CLL/RT, with promising results (108). Interestingly, one patient with RT experienced CR from his transformed component but persistence of the CLL counterpart. No major toxic effect and/or graft-versus-host disease was reported. Despite the HLA mismatch, CAR-NK cells were found to persist at low levels after 12 months from infusion.

## Treatment of HL-RT

The standard of care for *de novo* HL is the regimen indicated for patients with the HL-RT (109–112), with a reported response rate of 40-60% under ABVD (Doxorubicin, Bleomycin, Vinblastine, Dacarbazine). The median OS is 4 years in this setting. Bleomycin exposure can cause a severe pulmonary toxicity, leading to investigate the omission of this agent from the standard ABVD regimen (112). Following the results coming from the setting of advanced HLs, bleomycin can be safely omitted after two cycles of ABVD if interim PET shows remission (Deauville score 1–3). Escalation to BEACOPP in fit and younger patients should be considered in case of a positive interim PET, while radiotherapy could be an option for older and unfit patients (113). Stem cell transplantation is less used for consolidation in this setting, because of the longer survival observed compared to the DLBCL variants.

## Future Perspectives

### Diagnosis

Artificial intelligence tools can assist the diagnostic process for patients with a suspected RT. Four biomarkers have been recently identified to have consistent value for an RT-diagnosis model, according to cytologic (nuclear size and nuclear intensity) and architectural (cellular density and cell to nearest-neighbor distance) characteristics (114). This model was used to distinguish CLL from aCLL and RT cases with a good performance, and could be of support for further studies. Given the importance of distinguishing between aCLL and RT to select the correct therapeutic approach, more efforts to define a biological picture underlying the proliferation of RT cells are of outmost value in the era of targeted therapies.

PET/CT parameters SUV-related (i.e., SUV lean body mass, SUV body surface area, lesion-to-liver SUV ratio, and lesion-to-blood-pool SUV ratio) showed a correlation with DLBCL-RT diagnosis and/or OS and represent possible candidates for diagnostic biomarkers to further explore (115, 116). Moreover, novel PET radiotracers and PET–MRI are being explored in the setting of RT (117).

### Biology and Treatment

CDK4/6 inhibitors (i.e., palbociclib) have been recently identified as potential agents to overcome *CDKN2A/B* dysregulation (40). Palbociclib demonstrated activity in inhibiting RT-cell proliferation and showed an *in vitro* synergistic activity when combined with the BCR-signaling directed compounds ibrutinib, idelalisib, and fostamatinib.

*LAG3* is an emerging target for immune checkpoint blockade (50). Clinical trials are investigating *LAG3* inhibitors in hematological and solid cancers (NCT02061761; NCT01968109). Further assessment of *LAG3* inhibition, either alone or in combination with anti-PD-1 to enhance anti-tumor T-cell responses in RT is warranted.

Genomic data from the WGS confirm the pathogenic role of DNA damage response (DDR) pathway deregulation in RT (45). The role of DDR inhibitors such as PARP or ATR inhibitors has still to be assessed in RT.

The antibody-conjugate VLS-101 includes a humanized immunoglobulin G1 monoclonal antibody that binds ROR1, which is expressed by CLL lymphocytes to regulate chemotaxis and proliferation signaling (118, 119). VLS-101 attained complete and sustained remissions in RT patient-derived xenografts (RT-PDXs) expressing high levels of ROR1 (120). A phase 1 clinical trial of VLS-101 (NCT03833180) is enrolling patients with RT and other hematological neoplasms. Concomitantly, a phase 1 clinical trial (NCT02706392) is exploring the efficacy of anti-ROR1 CAR-T cells in patients with refractory CLL.

U-RT1, is a cell line derived from a highly proliferating RT clonally related to the underlying CLL (121). It is characterized by a complex karyotype with driver aberrations characteristic for RT such as genetic alterations of *TP53*, *CDKN2A*, and *NOTCH1*. This model represents a valuable tool for RT investigations and drug development.

Data on three newly established PDX models of RT-DLBCLs were recently published, namely, clonally-related and clonally-unrelated RT (122). These PDX models display protein expression of IRF4, TCF4, and BCL2. CRISPR knockout of *IRF4* led to reduced c-Myc levels and increased sensitivity to BET inhibitors. Co-treatment with a BET inhibitor or BET-PROTAC and ibrutinib or venetoclax showed synergistic *in vitro* lethality in the RT-DLBCL cells. When compared to single agent, combination of BET-PROTAC and venetoclax significantly reduced tumor burden and improved survival in immune-depleted mice engrafted with clonally related RT-DLBCL.

A potential synergistic effect of PI3K and BCL2 inhibitors has been proposed, based on the crosstalk between PI3K and apoptotic pathways (123). It has been shown that the inhibition of PI3K signaling by duvelisib leads to GSK3b activation and subsequent degradation of both c-Myc and Mcl-1. This crosstalk sensitizes RT cells to BCL-2 inhibition. Drug combination trials are ongoing, also in the setting of RT-DLBCL (NCT03892044).

In the field of CARs, targeting the transmembrane protein CD37 is another potential application for patients with B-cell malignancies. CD37 is expressed in mature B cells and at lower levels also on plasma cells and dendritic cells. Indeed, CD37 CAR-T cells were found to play a cytotoxic activity *in vivo* in B-cell tumor models (124). Dual targeting has already been suggested as a method to overcome treatment resistance due to the development of specific antigen loss consequent to CAR infusion. A bispecific CD37/CD19 CAR-T product is being developed to assess safety and efficacy in preclinical B-cell tumor models. Bispecific CD19/22 CAR-T cells have been already explored in non-Hodgkin lymphomas (NCT03196830), showing promising results (ORR 79.3%, CR 34.5% with 12-month PFS and OS of 40 and 63%, respectively) (125). The employment of CD19 CAR-NK cells in B-cell malignancies is also being explored in different ongoing phase 1 trials (i.e., NCT04887012, NCT04639739, NCT04796675, and NCT05020678), and novel targets for CAR-NK cells are object of study (i.e., CAR70/IL15-transduced NK cells in NCT05092451). Efficacy of these agents needs to be assessed in the setting of RT.

A list of ongoing trials with targeted agents in RT is reported in **Table 2** (updated from clinicaltrials.gov on Feb 20, 2022).

## Conclusions

Patients with CLL progressing on novel agents represent a new high-risk prognostic group with adverse outcome in case of transformation. The promising combination of CIT with the novel agent venetoclax for DLBCL-RT confirms the synergistic effect of the approaches. The availability of new preclinical models is progressively expanding our understanding of RT biology, laying the foundations for targeted treatments which might be better tolerated.

## Author Contributions

AC and DR wrote the manuscript. All authors listed have made a substantial, direct, and intellectual contribution to the work and approved it for publication.

## Conflict of Interest

The authors declare that the research was conducted in the absence of any commercial or financial relationships that could be construed as a potential conflict of interest.

## Publisher’s Note

All claims expressed in this article are solely those of the authors and do not necessarily represent those of their affiliated organizations, or those of the publisher, the editors and the reviewers. Any product that may be evaluated in this article, or claim that may be made by its manufacturer, is not guaranteed or endorsed by the publisher.

## References

[1] Swerdlow SH Campo E Harris NL Jaffe ES Pileri SA Stein H. WHO Classification of Tumours of Haematopoietic and Lymphoid Tissues. 4th Ed. Lyon: International Agency for Research on Cancer (2017).

[2] Richter MN. Generalized Reticular Cell Sarcoma of Lymph Nodes Associated With Lymphatic Leukemia. Am J Pathol (1928) 4:285–92. PMC2006994 19969796

[3] Lortholary P Boiron M Ripault P Levy JP Manus A Bernard J. Chronic Lymphoid Leukemia Secondarily Associated With a Malignant Reticulopathy: Richter's Syndrome. Nouv Rev Fr Hematol (1964) 4:621–44. 14199493

[4] Parikh SA Rabe KG Call TG Zent CS Habermann TM Ding W. Diffuse Large B-Cell Lymphoma (Richter Syndrome) in Patients With Chronic Lymphocytic Leukaemia (CLL): A Cohort Study of Newly Diagnosed Patients. Br J Haematol (2013) 162:774–82. doi: 10.1111/bjh.12458 PMC4098845 23841899

[5] Marvyin K Tjønnfjord EB Breland UM Tjønnfjord GE. Transformation to Plasmablastic Lymphoma in CLL Upon Ibrutinib Treatment. BMJ Case Rep (2020) 13:e235816. doi: 10.1136/bcr-2020-235816 PMC7526319 32994268

[6] Abrisqueta P Delgado J Alcoceba M Oliveira AC Loscertales J Hernández-Rivas JA. Clinical Outcome and Prognostic Factors of Patients With Richter Syndrome: Real-World Study of the Spanish Chronic Lymphocytic Leukemia Study Group (GELLC). Br J Haematol (2020) 190(6):854–63. doi: 10.1111/bjh.16748 32519351

[7] Lenartova A Randen U Johannesen TB Tjønnfjord GE. Richter Syndrome Epidemiology in a Large Population Based Chronic Lymphocytic Leukemia Cohort From Norway. Cancer Epidemiol (2019) 60:128e33. doi: 10.1016/j.canep.2019.04.002 30986631

[8] Agbay RL Jain N Loghavi S Medeiros LJ Khoury JD. Histologic Transformation of Chronic Lymphocytic Leukemia/Small Lymphocytic Lymphoma. Am J Hematol (2016) 91(10):1036e43. doi: 10.1002/ajh.24473 27414262

[9] Al-Sawaf O Robrecht S Bahlo J Fink AM Cramer P V Tresckow J. Richter Transformation in Chronic Lymphocytic Leukemia (CLL)-A Pooled Analysis of German CLL Study Group (GCLLSG) Front Line Treatment Trials. Leukemia (2020) 35(1):169–76. doi: 10.1038/s41375-020-0797-x 32203141

[10] Elnair R Ellithi M Kallam A Shostrom V Bociek RG. Outcomes of Richter's Transformation of Chronic Lymphocytic Leukemia/Small Lymphocytic Lymphoma (CLL/SLL): An Analysis of the SEER Database. Ann Hematol (2021) 100(10):2513–9. doi: 10.1007/s00277-021-04603-y 34279675

[11] Rossi D Berra E Cerri M Deambrogi C Barbieri C Franceschetti S. Aberrant Somatic Hypermutation in Transformation of Follicular Lymphoma and Chronic Lymphocytic Leukemia to Diffuse Large B-Cell Lymphoma. Haematologica (2006) 91:1405–9. doi: 10.3324/%x 17018394

[12] Parikh SA Habermann TM Chaffee KG Call TG Ding W Leis JF. Hodgkin Transformation of Chronic Lymphocytic Leukemia: Incidence, Outcomes, and Comparison to *De Novo* Hodgkin Lymphoma. Am J Hematol (2015) 90(4):334–8. doi: 10.1002/ajh.23939 PMC4438308 25581025

[13] Ahn IE Underbayev C Albitar A Herman SEM Tian X Maric I. Clonal Evolution Leading to Ibrutinib Resistance in Chronic Lymphocytic Leukemia. Blood (2017) 129:1469–79. doi: 10.1182/blood-2016-06-719294 PMC5356450 28049639

[14] Burger JA Tedeschi A Barr PM Robak T Owen C Ghia P. Ibrutinib as Initial Therapy for Patients With Chronic Lymphocytic Leukemia. N Engl J Med (2015) 373:2425–37. doi: 10.1056/NEJMoa1509388 PMC4722809 26639149

[15] Woyach JA Ruppert AS Heerema NA Zhao W Booth AM Ding W. Ibrutinib Regimens Versus Chemoimmunotherapy in Older Patients With Untreated CLL. N Engl J Med (2018) 379:2517–28. doi: 10.1056/NEJMoa1812836 PMC6325637 30501481

[16] Moreno C Greil R Demirkan F Tedeschi A Anz B Larratt L. Ibrutinib Plus Obinutuzumab Versus Chlorambucil Plus Obinutuzumab in First-Line Treatment of Chronic Lymphocytic Leukaemia (iLLUMINATE): A Multicentre, Randomised, Open-Label, Phase 3 Trial. Lancet Oncol (2019) 20:43–56. doi: 10.1016/S1470-2045(18)30788-5 30522969

[17] Shanafelt TD Wang XV Kay NE Hanson CA O’Brien S Barrientos J. Ibrutinib–Rituximab or Chemoimmunotherapy for Chronic Lymphocytic Leukemia. N Engl J Med (2019) 381:432–43. doi: 10.1056/NEJMoa1817073 PMC6908306 31365801

[18] Sharman JP Egyed M Jurczak W Skarbnik A Pagel JM Flinn IW. Acalabrutinib With or Without Obinutuzumab Versus Chlorambucil and Obinutuzmab for Treatment-Naive Chronic Lymphocytic Leukaemia (ELEVATE TN): A Randomised, Controlled, Phase 3 Trial. Lancet (2020) 395:1278–91. doi: 10.1016/S0140-6736(20)30262-2 PMC8151619 32305093

[19] O’Brien SM Lamanna N Kipps TJ Flinn I Zelenetz AD Burger JA. A Phase 2 Study of Idelalisib Plus Rituximab in Treatment-Naïve Older Patients With Chronic Lymphocytic Leukemia. Blood (2015) 126:2686–94. doi: 10.1182/blood-2015-03-630947 PMC4732760 26472751

[20] Lampson BL Kim HT Davids MS Abramson JS Freedman AS Jacobson CA. Efficacy Results of a Phase 2 Trial of First-Line Idelalisib Plus Ofatumumab in Chronic Lymphocytic Leukemia. Blood Adv (2019) 3:1167–74. doi: 10.1182/bloodadvances.2018030221 PMC6457234 30967392

[21] Fischer K Al-Sawaf O Bahlo J Fink A-M Tandon M Dixon M. Venetoclax and Obinutuzumab in Patients With CLL and Coexisting Conditions. N Engl J Med (2019) 380:2225–36. doi: 10.1056/NEJMoa1815281 31166681

[22] Kadri S Lee J Fitzpatrick C Galanina N Sukhanova M Venkataraman G. Clonal Evolution Underlying Leukemia Progression and Richter Transformation in Patients With Ibrutinib-Relapsed CLL. Blood Adv (2017) 1:715–27. doi: 10.1182/bloodadvances.2016003632 PMC5728051 29296715

[23] Vaisitti T Braggio E Allan JN Arruga F Serra S Zamò A. Novel Richter Syndrome Xenograft Models to Study Genetic Architecture, Biology, and Therapy Responses. Cancer Res (2018) 78:3413–20. doi: 10.1158/0008-5472.CAN-17-4004 29735551

[24] Bruzzi JF Macapinlac H Tsimberidou AM Truong MT Keating MJ Marom EM. Detection of Richter's Transformation of Chronic Lymphocytic Leukemia by PET/Ct. J Nucl Med (2006) 47:1267–73. 16883004

[25] Gascoyne RD XIV. The Pathology of Transformation of Indolent B Cell Lymphomas. Hematol Oncol (2015) 33 (Suppl 1):75–9. doi: 10.1002/hon.2222 26062060

[26] Falchi L Keating MJ Marom EM Truong MT Schlette EJ Sargent RL. Correlation Between FDG/PET, Histology, Characteristics, and Survival in 332 Patients With Chronic Lymphoid Leukemia. Blood (2014) 123(18):2783–90. doi: 10.1182/blood-2013-11-536169 PMC4123418 24615780

[27] Mauro FR Chauvie S Paoloni F Biggi A Cimino G Rago A. Diagnostic and Prognostic Role of PET/CT in Patients With Chronic Lymphocytic Leukemia and Progressive Disease. Leukemia (2015) 29(6):1360–5. doi: 10.1038/leu.2015.21 25650091

[28] Michallet AS Sesques P Rabe KG Itti E Tordot J Tychyj-Pinel C. An 18f-FDG-PET Maximum Standardized Uptake Value > 10 Represents a Novel Valid Marker for Discerning Richter's Syndrome. Leuk Lymphoma (2016) 57:1474–7. doi: 10.3109/10428194.2015.1099643 26402256

[29] Mato AR Wierda WG Davids MS Cheson BD Coutre SE Choi M. Analysis of PET-CT to Identify Richter’s Transformation in 167 Patients With Disease Progression Following Kinase Inhibitor Therapy. Blood (2017) 130(suppl 1):Abstract 834. doi: 10.1182/blood.V130.Suppl_1.834.834

[30] Rossi D Spina V Deambrogi C Rasi S Laurenti L Stamatopoulos K. The Genetics of Richter Syndrome Reveals Disease Heterogeneity and Predicts Survival After Transformation. Blood (2011) 117:3391–401. doi: 10.1182/blood-2010-09-302174 21266718

[31] Soilleux EJ Wotherspoon A Eyre TA Clifford R Cabes M Schuh AH. Diagnostic Dilemmas of High-Grade Transformation (Richter's Syndrome) of Chronic Lymphocytic Leukaemia: Results of the Phase II National Cancer Research Institute CHOP-OR Clinical Trial Specialist Haemato-Pathology Central Review. Histopathology (2016) 69:1066–76. doi: 10.1111/his.13024 27345622

[32] Giné E Martinez A Villamor N López-Guillermo A Camos M Martinez D. Expanded and Highly Active Proliferation Centers Identify a Histological Subtype of Chronic Lymphocytic Leukemia ("Accelerated" Chronic Lymphocytic Leukemia) With Aggressive Clinical Behavior. Haematologica (2010) 95:1526–33. doi: 10.3324/haematol.2010.022277 PMC2930954 20421272

[33] Rossi D Spina V Cerri M Rasi S Deambrogi C De Paoli L. Stereotyped B-Cell Receptor is an Independent Risk Factor of Chronic Lymphocytic Leukemia Transformation to Richter Syndrome. Clin Cancer Res (2009) 15:4415–22. doi: 10.1158/1078-0432.CCR-08-3266 19509140

[34] Mao Z Quintanilla-Martinez L Raffeld M Richter M Krugmann J Burek C. IgVH Mutational Status and Clonality Analysis of Richter’s Transformation. Am J Surg Pathol (2007) 31:1605–14. doi: 10.1097/PAS.0b013e31804bdaf8 17895764

[35] He R Ding W Viswanatha DS Chen D Shi M Van Dyke D. PD-1 Expression in Chronic Lymphocytic Leukemia/Small Lymphocytic Lymphoma (CLL/SLL) and Large B-Cell Richter Transformation (DLBCL-RT): A Characteristic Feature of DLBCL-RT and Potential Surrogate Marker for Clonal Relatedness. Am J Surg Pathol (2018) 42(7):843–54. doi: 10.1097/PAS.0000000000001077 29762141

[36] Behdad A Griffin B Chen YH Ma S Kelemen K Lu X. PD-1 is Highly Expressed by Neoplastic B-Cells in Richter Transformation. Br J Haematol (2019) 185(2):370–3. doi: 10.1111/bjh.15514 30028010

[37] Fabbri G Khiabanian H Holmes AB Wang J Messina M Mullighan CG. Genetic Lesions Associated With Chronic Lymphocytic Leukemia Transformation to Richter Syndrome. J Exp Med (2013) 210:2273–88. doi: 10.1084/jem.20131448 PMC3804949 24127483

[38] Chigrinova E Rinaldi A Kwee I Rossi D Rancoita PM Strefford JC. Two Main Genetic Pathways Lead to the Transformation of Chronic Lymphocytic Leukemia to Richter Syndrome. Blood (2013) 122:2673–782. doi: 10.1182/blood-2013-03-489518 24004666

[39] De Paoli L Cerri M Monti S Rasi S Spina V Bruscaggin A. MGA, a Suppressor of MYC, Is Recurrently Inactivated in High Risk Chronic Lymphocytic Leukemia. Leuk Lymphoma (2013) 54:1087–90. doi: 10.3109/10428194.2012.723706 23039309

[40] Chakraborty S Martines C Porro F Fortunati I Bonato A Dimishkovska M. B-Cell Receptor Signaling and Genetic Lesions in TP53 and CDKN2A/CDKN2B Cooperate in Richter Transformation. Blood (2021) 138(12):1055–68. doi: 10.1182/blood.2020008276 33900379

[41] Gounari M Ntoufa S Apollonio B Papakonstantinou N Ponzoni M Chu CC. Excessive Antigen Reactivity may Underlie the Clinical Aggressiveness of Chronic Lymphocytic Leukemia Stereotyped Subset 8. Blood (2015) 125(23):3580–7. doi: 10.1182/blood-2014-09-603217 PMC4458798 25900981

[42] Rossi D Rasi S Fabbri G Spina V Fangazio M Forconi F. Mutations of NOTCH1 Are an Independent Predictor of Survival in Chronic Lymphocytic Leukemia. Blood (2012) 119:521–9. doi: 10.1182/blood-2011-09-379966 PMC3257017 22077063

[43] Rossi D Rasi S Spina V Fangazio M Monti S Greco M. Different Impact of NOTCH1and SF3B1 Mutations on the Risk of Chronic Lymphocytic Leukemia Transformation to Richter Syndrome. Br J Haematol (2012) 158:426–9. doi: 10.1111/j.1365-2141.2012.09155.x 22571487

[44] Villamor N Conde L Martínez-Trillos A Cazorla M Navarro A Beà S. NOTCH1 Mutations Identify a Genetic Subgroup of Chronic Lymphocytic Leukemia Patients With High Risk of Transformation and Poor Outcome. Leukemia (2013) 27:1100–6. doi: 10.1038/leu.2012.357 23295735

[45] Klintman J Appleby N Stamatopoulos B Ridout K Eyre TA Robbe P. Genomic and Transcriptomic Correlates of Richter Transformation in Chronic Lymphocytic Leukemia. Blood (2021) 137(20):2800–16. doi: 10.1182/blood.2020005650 PMC8163497 33206936

[46] Wang Y Sinha S Wellik LE Secreto CR Rech KL Call TG. Distinct Immune Signatures in Chronic Lymphocytic Leukemia and Richter Syndrome. Blood Cancer J (2021) 11(5):86. doi: 10.1038/s41408-021-00477-5 33972504 PMC8110984

[47] Ding W LaPlant BR Call TG Parikh SA Leis JF He R. Pembrolizumab in Patients With CLL and Richter Transformation or With Relapsed CLL. Blood (2017) 129(26):3419–27. doi: 10.1182/blood-2017-02-765685 PMC5492091 28424162

[48] Teng MW Ngiow SF Ribas A Smyth MJ. Classifying Cancers Based on T-Cell Infiltration and PD-L1. Cancer Res (2015) 75:2139–45. doi: 10.1158/0008-5472.CAN-15-0255 PMC4452411 25977340

[49] Smyth MJ Ngiow SF Ribas A Teng MW. Combination Cancer Immunotherapies Tailored to the Tumour Microenvironment. Nat Rev Clin Oncol (2016) 13:143–58. doi: 10.1038/nrclinonc.2015.209 26598942

[50] Gould C Lickiss J Kankanige Y Yerneni S Lade S Gandhi MK. Characterisation of Immune Checkpoints in Richter Syndrome Identifies LAG3 as a Potential Therapeutic Target. Br J Haematol (2021) 195(1):113–8. doi: 10.1111/bjh.17789 34426978

[51] Tobin JWD Keane C Gunawardana J Mollee P Birch S Hoang T. Progression of Disease Within 24 Months in Follicular Lymphoma is Associated With Reduced Intratumoral Immune Infiltration. J Clin Oncol (2019) 37(34):3300–9. doi: 10.1200/JCO.18.02365 PMC6881104 31461379

[52] Tumeh PC Harview CL Yearley JH Shintaku IP Taylor EJM Robert L. PD-1 Blockade Induces Responses by Inhibiting Adaptive Immune Resistance. Nature (2014) 515(7528):568–71. doi: 10.1038/nature13954 PMC4246418 25428505

[53] Kohlhaas V Blakemore SJ Al-Maarri M Nickel N Pal M Roth A. Active Akt Signaling Triggers CLL Toward Richter Transformation *via* Overactivation of Notch1. Blood (2021) 137(5):646–60. doi: 10.1182/blood.2020005734 33538798

[54] Nagasawa T Hirota S Tachibana K Takakura N Nishikawa S Kitamura Y. Defects of B-Cell Lymphopoiesis and Bone-Marrow Myelopoiesis in Mice Lacking the CXC Chemokine PBSF/SDF-1. Nature (1996) 382:635–8. doi: 10.1038/382635a0 8757135

[55] Ding L Morrison SJ. Haematopoietic Stem Cells and Early Lymphoid Progenitors Occupy Distinct Bone Marrow Niches. Nature (2013) 495:231–5. doi: 10.1038/nature11885 PMC3600153 23434755

[56] Burger JA Tsukada N Burger M Zvaifler NJ Dell’Aquila M Kipps TJ. Blood-Derived Nurse-Like Cells Protect Chronic Lymphocytic Leukemia B Cells From Spontaneous Apoptosis Through Stromal Cell-Derived Factor-1. Blood (2000) 96:2655–63. doi: 10.1182/blood.V96.8.2655 11023495

[57] Mohle R Failenschmid C Bautz F Kanz L. Overexpression of the Chemokine Receptor CXCR4 in B Cell Chronic Lymphocytic Leukemia is Associated With Increased Functional Response to Stromal Cell-Derived Factor-1 (SDF-1). Leukemia (1999) 13:1954–9. doi: 10.1038/sj.leu.2401602 10602415

[58] Lewis R Maurer HC Singh N Gonzalez-Menendez I Wirth M Schick M. CXCR4 Hyperactivation Cooperates With TCL1 in CLL Development and Aggressiveness. Leukemia (2021) 35(10):2895–905. doi: 10.1038/s41375-021-01376-1 PMC8478649 34363012

[59] Tsimberidou A-M O’Brien S Khouri I Giles FJ Kantarjian HM Champlin R. Clinical Outcomes and Prognostic Factors in Patients With Richter’s Syndrome Treated With Chemotherapy or Chemoimmunotherapy With or Without Stem-Cell Transplantation. J Clin Oncol (2006) 24:2343–51. doi: 10.1200/JCO.2005.05.0187 16710033

[60] Visentin A Bonaldi L Rigolin GM Mauro FR Martines A Frezzato F. The Complex Karyotype Landscape in Chronic Lymphocytic Leukemia Allows to Refine the Risk of Richter Syndrome Transformation. Haematologica (2021). doi: 10.3324/haematol.2021.278304 PMC8968897 34092056

[61] Jain P Thompson PA Keating M Estrov Z Ferrajoli A Jain N. Longterm Outcomes for Patients With Chronic Lymphocytic Leukemia Who Discontinue Ibrutinib. Cancer (2017) 123(12):2268–73. doi: 10.1002/cncr.30596 PMC5980235 28171709

[62] Byrd JC Blum KA Burger JA Coutre SE Sharman JP Furman RR. Activity and Tolerability of the Bruton’s Tyrosine Kinase (Btk) Inhibitor PCI-32765 in Patients With Chronic Lymphocytic Leukemia/Small Lymphocytic Lymphoma (CLL/SLL): Interim Results of a Phase Ib/II Study [Abstract]. J Clin Oncol (2011) 29(suppl 15):6508. doi: 10.1200/jco.2011.29.15_suppl.6508

[63] Davids MS Huang Y Rogers KA Stern R Brown JR Thompson PA. Richter’s Syndrome (RS) in Patients With Chronic Lymphocytic Leukemia (CLL) on Novel Agent Therapy [Abstract]. J Clin Oncol (2017) 35(suppl 15):7505. doi: 10.1200/JCO.2017.35.15_suppl.7505

[64] Anderson MA Tam C Lew TE Juneja S Juneja M Westerman D. Clinicopathological Features and Outcomes of Progression of CLL on the BCL2 Inhibitor Venetoclax. Blood (2017) 129:3362–70. doi: 10.1182/blood-2017-01-763003 28473407

[65] Mato AR Nabhan C Barr PM Ujjani CS Hill BT Lamanna N. Outcomes of CLL Patients Treated With Sequential Kinase Inhibitor Therapy: A Real World Experience. Blood (2016) 128:2199–205. doi: 10.1182/blood-2016-05-716977 27601462

[66] Lew TE Lin VS Cliff ER Blombery P Thompson ER Handunnetti SM. Outcomes of Patients With CLL Sequentially Resistant to Both BCL2 and BTK Inhibition. Blood Adv (2021) 5(20):4054–8. doi: 10.1182/bloodadvances.2021005083 PMC8945613 34478505

[67] Langerbeins P Busch R Anheier N Dürig J Bergmann M Goebeler ME. Poor Efficacy and Tolerability of R-CHOP in Relapsed/Refractory Chronic Lymphocytic Leukemia and Richter Transformation. Am J Hematol (2014) 89:E239–243. doi: 10.1002/ajh.23841 25196783

[68] Wierda WG Kipps TJ Mayer J Stilgenbauer S Williams CD Hellmann A. Ofatumumab as Single-Agent CD20 Immunotherapy in fludarabine-Refractory Chronic Lymphocytic Leukemia. J Clin Oncol (2010) 28:1749–55. doi: 10.1200/JCO.2009.25.3187 PMC4979101 20194866

[69] Eyre TA Clifford R Bloor A Boyle L Roberts C Cabes M. NCRI Phase II Study of CHOP in Combination With Ofatumumab in Induction and Maintenance in Newly Diagnosed Richter Syndrome. Br J Haematol (2016) 175:43–54. doi: 10.1111/bjh.14177 27378086

[70] Rogers KA Salem G Stephens DM Andritsos LA Awan FT Byrd JC. A Single-Institution Retrospective Cohort Study of Patients Treated With R-EPOCH for Richter's Transformation of Chronic Lymphocytic Leukemia. Blood (2015) 126:2951. doi: 10.1182/blood.V126.23.2951.2951

[71] Dabaja BS O’Brien SM Kantarjian HM Cortes JE Thomas DA Albitar M. Fractionated Cyclophosphamide, Vincristine, Liposomal Daunorubicin (Daunoxome), and Dexamethasone (Hyper-CVXD) Regimen in Richter’s Syndrome. Leuk Lymphoma (2001) 42:329–37. doi: 10.3109/10428190109064589 11699397

[72] Tsimberidou AM Kantarjian HM Cortes J Thomas DA Faderl S Garcia-Manero G. Fractionated Cyclophosphamide, Vincristine, Liposomal Daunorubicin, and Dexamethasone Plus Rituximab and Granulocytemacrophage- Colony Stimulating Factor (GM-CSF) Alternating With Methotrexate and Cytarabine Plus Rituximab and GMCSF in Patients With Richter Syndrome or Fludarabine Refractory Chronic Lymphocytic Leukemia. Cancer (2003) 97:1711–20. doi: 10.1002/cncr.11238 12655528

[73] Tsimberidou AM Wierda WG Plunkett W Kurzrock R O’Brien S Wen S. Phase I-II Study of Oxaliplatin, Fludarabine, Cytarabine, and Rituximab Combination Therapy in Patients With Richter’s Syndrome or Fludarabine-Refractory Chronic Lymphocytic Leukemia. J Clin Oncol (2008) 26:196–203. doi: 10.1200/JCO.2007.11.8513 18182662

[74] Tsimberidou AM Wierda WG Wen S Plunkett W O'Brien S Kipps TJ. Phase I-II Clinical Trial of Oxaliplatin, Fludarabine, Cytarabine, and Rituximab Therapy in Aggressive Relapsed/Refractory Chronic Lymphocytic Leukemia or Richter Syndrome. Clin Lymphoma Myeloma Leuk (2013) 13:568–74. doi: 10.1016/j.clml.2013.03.012 PMC4180513 23810245

[75] Cwynarski K van Biezen A de Wreede L Stilgenbauer S Bunjes D Metzner B. Autologous and Allogeneic Stem-Cell Transplantation for Transformed Chronic Lymphocytic Leukemia (Richter's Syndrome): A Retrospective Analysis From the Chronic Lymphocytic Leukemia Subcommittee of the Chronic Leukemia Working Party and Lymphoma Working Party of the European Group for Blood and Marrow Transplantation. J Clin Oncol (2012) 30:2211–7. doi: 10.1200/JCO.2011.37.4108 22547610

[76] Herrera AF Ahn KW Litovich C Chen Y Assal A Bashir Q. Autologous and Allogeneic Hematopoietic Cell Transplantation for Diffuse Large B-Cell Lymphoma-Type Richter Syndrome. Blood Adv (2021) 5(18):3528–39. doi: 10.1182/bloodadvances.2021004865 PMC8945575 34496026

[77] Lahoud OB Devlin SM Maloy MA Roeker LE Dahi PB Ponce DM. Reduced-Intensity Conditioning Hematopoietic Stem Cell Transplantation for Chronic Lymphocytic Leukemia and Richter's Transformation. Blood Adv (2021) 5(14):2879–89. doi: 10.1182/bloodadvances.2020003726 PMC8341347 34297048

[78] Aulakh S Reljic T Yassine F Ayala E Chavez JC Chanan-Khan A. Allogeneic Hematopoietic Cell Transplantation is an Effective Treatment for Patients With Richter Syndrome: A Systematic Review and Meta-Analysis. Hematol Oncol Stem Cell Ther (2021)14(1):33–40. doi: 10.1016/j.hemonc.2020.05.002. 32473105 PMC7666647

[79] Kuruvilla J Byrd JC Flynn JM Garzon R Porcu P Wagner-Johnston N. The Oral Selective Inhibitor of Nuclear Export (SINE) Selinexor (KPT-330) Demonstrates Broad and Durable Clinical Activity in Relapsed/Refractory non Hodgkin’s Lymphoma (NHL). Blood (ASH Annu Meet Abstracts) (2014) 124:396. doi: 10.1182/blood.V124.21.396.396

[80] Tsang M Shanafelt TD Call TG Ding W Chanan-Khan A Leis JF. The Efficacy of Ibrutinib in the Treatment of Richter Syndrome. Blood (2015) 125:1676–8. doi: 10.1182/blood-2014-12-610782 PMC4351511 25745187

[81] Giri S Hahn A Yaghmour G Martin MG. Ibrutinib has Some Activity in Richter's Syndrome. Blood Cancer J (2015) 5(1):e277. doi: 10.1038/bcj.2014.98 25635530 PMC5404220

[82] Master S Leary C Takalkar A Coltelingam J Mansour R Mills GM. Successful Treatment of Richter Transformation With Ibrutinib in a Patient With Chronic Lymphocytic Leukemia Following Allogeneic Hematopoietic Stem Cell Transplant. Case Rep Oncol (2017) 10(2):534–41. doi: 10.1159/000477338 PMC5498945 28690529

[83] Hampel PJ Cherng HJ Call TG Ding W Khanlari M McPhail ED. Incidental Richter Transformation in Chronic Lymphocytic Leukemia Patients During Temporary Interruption of Ibrutinib. Blood Adv (2020) 4(18):4508–11. doi: 10.1182/bloodadvances.2020002454 PMC7509865 32946567

[84] Eyre TA Schuh A Wierda WG Brown JR Ghia P Pagel JM. Acalabrutinib Monotherapy for Treatment of Chronic Lymphocytic Leukaemia (ACE-CL-001): Analysis of the Richter Transformation Cohort of an Open-Label, Single-Arm, Phase 1-2 Study. Lancet Haematol (2021) 8(12):e912–21. doi: 10.1016/S2352-3026(21)00305-7 PMC11729767 34735860

[85] Mato AR Shah NN Lamanna N Eyre TA Jurczak W Woyach JA. Pirtobrutinib (LOXO-305), a Next Generation, Highly Selective, Non-Covalent BTK Inhibitor in Previously Treated Richter Transformation: Results From the Phase 1/2 BRUIN Study. . HemaSphere (2021) 5(S2):226–7. doi: 10.1097/HS9.0000000000000566

[86] Reiff SD Mantel R Smith LL Greene JT Muhowski EM Fabian CA. The BTK Inhibitor ARQ 531 Targets Ibrutinib-Resistant CLL and Richter Transformation. Cancer Discovery (2018) 8(10):1300–15. doi: 10.1158/2159-8290.CD-17-1409 PMC6261467 30093506

[87] Lucas F KA R BK H Pan A Yu L Breitbach J. Eμ-TCL1xMyc: A Novel Mouse Model for Concurrent CLL and B-Cell Lymphoma. Clin Cancer Res (2019) 25(20):6260–73. doi: 10.1158/1078-0432.CCR-19-0273 PMC6801062 31296529

[88] Kohlhaas V Blakemore SJ Al-Maarri M Nickel N Pal M Roth A. Active Akt signaling triggers CLL toward Richter transformation *via* overactivation of Notch1. Blood (2021) 137(5):646–60. doi: 10.1182/blood.2020005734 33538798

[89] Visentin A Imbergamo S Scomazzon E Pravato S Frezzato F Bonaldi L. BCR Kinase Inhibitors, Idelalisib and Ibrutinib, are Active and Effective in Richter Syndrome. Br J Haematol (2019) 185(1):193–7. doi: 10.1111/bjh.15440 29974955

[90] Davids MS Roberts AW Seymour JF Pagel JM Kahl BS Wierda WG. Phase I First-In-Human Study of Venetoclax in Patients With Relapsed or Refractory Non-Hodgkin Lymphoma. J Clin Oncol (2017) 35(8):826–33. doi: 10.1200/JCO.2016.70.4320 PMC5455685 28095146

[91] Davids MS Rogers KA Tyekucheva S Wang Z Pazienza S Renner SK. Venetoclax Plus Dose-Adjusted R-EPOCH (VR-EPOCH) for Richter's Syndrome. Blood (2022) 139(5):686–9. doi: 10.1182/blood.2021011386. PMC8814674 34788401

[92] Sagiv-Barfi I Kohrt HE Czerwinski DK Ng PP Chang BY Levy R. Therapeutic Antitumor Immunity by Checkpoint Blockade is Enhanced by Ibrutinib, an Inhibitor of Both BTK and ITK. Proc Natl Acad Sci USA (2015) 112:E966–72. doi: 10.1073/pnas.1500712112 PMC4352777 25730880

[93] Younes A Brody J Carpio C Lopez-Guillermo A Ben-Yehuda D Ferhanoglu B. Safety and Activity of Ibrutinib in Combination With Nivolumab in Patients With Relapsed non-Hodgkin Lymphoma or Chronic Lymphocytic Leukaemia: A Phase 1/2a Study. Lancet Haematol (2019) 6(2):e67–78. doi: 10.1016/S2352-3026(18)30217-5 30642819

[94] Jain N Ferrajoli A Thompson PA Konopleva M Green MR Sampath D. Venetoclax, Obinutuzumab and Atezolizumab (PD-L1 Checkpoint Inhibitor) for Treatment for Patients With Richter Transformation. Blood (2021) 138(Supplement 1):1550. doi: 10.1182/blood-2021-154279

[95] Hutchings M Morschhauser F Iacoboni G Carlo-Stella C Offner FC Sureda A. Glofitamab, a Novel, Bivalent CD20-Targeting T-Cell-Engaging Bispecific Antibody, Induces Durable Complete Remissions in Relapsed or Refractory B-Cell Lymphoma: A Phase I Trial. J Clin Oncol (2021) 39(18):1959–70. doi: 10.1200/JCO.20.03175 PMC8210975 33739857

[96] Sato S Jansen PJ Tedder TF. CD19 and CD22 Expression Reciprocally Regulates Tyrosine Phosphorylation of Vav Protein During B Lymphocyte Signaling. Proc Natl Acad Sci USA (1997) 94:13158–62. doi: 10.1073/pnas.94.24.13158 PMC24279 9371816

[97] Scheuermann RH Racila E. CD19 Antigen in Leukemia and Lymphoma Diagnosis and Immunotherapy. Leuk Lymphoma (1995) 18:385–97. doi: 10.3109/10428199509059636 8528044

[98] Uckun F Jaszcz W Ambrus J Fauci AS Gajl-Peczalska K Song CW. Detailed Studies on Expression and Function of CD19 Surface Determinant by Using B43 Monoclonal Antibody and the Clinical Potential of Anti-CD19 Immunotoxins. Blood (1988) 71:13–29. doi: 10.1182/blood.V71.1.13.13 3257143

[99] Majzner RG Mackall CL. Tumor Antigen Escape From CAR T-Cell Therapy. Cancer Discovery (2018) 8:1219–26. doi: 10.1158/2159-8290.CD-18-0442 30135176

[100] Porter DL Hwang WT Frey NV Lacey SF Shaw PA Loren AW. Chimeric Antigen Receptor T Cells Persist and Induce Sustained Remissions in Relapsed Refractory Chronic Lymphocytic Leukemia. Sci Transl Med (2015) 7:303ra139. doi: 10.1126/scitranslmed.aac5415 PMC5909068 26333935

[101] Turtle CJ Hay KA Hanafi LA Li D Cherian S Chen X. Durable Molecular Remissions in Chronic Lymphocytic Leukemia Treated With CD19-Specific Chimeric Antigen Receptor-Modified T Cells After Failure of Ibrutinib. J Clin Oncol (2017) 35:3010–20. doi: 10.1200/JCO.2017.72.8519 PMC5590803 28715249

[102] Gauthier J Hirayama AV Purushe J Hay KA Lymp J Li DH. Feasibility and Efficacy of CD19-Targeted CAR T Cells With Concurrent Ibrutinib for CLL After Ibrutinib Failure. Blood (2020) 135:1650–60. doi: 10.1182/blood.2019002936 PMC7205814 32076701

[103] Benjamini O Shimoni A Besser M Shem-tov N Danylesko I Yerushalmi R. Safety and Efficacy of CD19-CAR T Cells in Richter’s Transformation After Targeted Therapy for Chronic Lymphocytic Leukemia. Blood (2020) 136(Suppl. S1):40. doi: 10.1182/blood-2020-138904

[104] Kittai AS Bond DA William B Saad A Penza S Efebera Y. Clinical Activity of Axicabtagene Ciloleucel in Adult Patients With Richter Syndrome. Blood Adv (2020) 4:4648–52. doi: 10.1182/bloodadvances.2020002783 PMC7556158 33002129

[105] Batlevi CL Palomba ML Park J Mead E Santomasso B Riviere I. Phase I Clinical Trial of CD19-Targeted 19-28Z/4-1BBL “Armored” CAR T Cells in Patients With Relapsed or Refractory NHL and CLL Including Richter Transformation. Hematol Oncol (2019) 37:166–7. doi: 10.1002/hon.124_2629

[106] Ortiz-Maldonado V Frigola G Español-Rego M Balagué O Martínez-Cibrián N Magnano L. Results of ARI-0001 CART19 Cells in Patients With Chronic Lymphocytic Leukemia and Richter's Transformation. Front Oncol (2022) 12:828471. doi: 10.3389/fonc.2022.828471 35174095 PMC8841853

[107] Shah N Li L McCarty J Kaur I Yvon E Shaim H. Phase I Study of Cord Blood-Derived Natural Killer Cells Combined With Autologous Stem Cell Transplantation in Multiple Myeloma. Br J Haematol (2017) 177:457–66. doi: 10.1111/bjh.14570 PMC5856008 28295190

[108] Liu E Marin D Banerjee P Macapinlac HA Thompson P Basar R. Use of CAR-Transduced Natural Killer Cells in CD19-Positive Lymphoid Tumors. N Engl J Med (2020) 382(6):545–53. doi: 10.1056/NEJMoa1910607 PMC7101242 32023374

[109] Tsimberidou AM O'Brien S Kantarjian HM Koller C Hagemeister FB Fayad L. Hodgkin Transformation of Chronic Lymphocytic Leukemia: The M. D. Anderson Cancer Center Experience. Cancer (2006) 107:1294–302. doi: 10.1002/cncr.22121 16902984

[110] Bockorny B Codreanu I and Dasanu CA. Hodgkin Lymphoma as Richter Transformation in Chronic Lymphocytic Leukaemia: A Retrospective Analysis of World Literature. Br J Haematol (2012) 156:50–66. doi: 10.1111/j.1365-2141.2011.08907.x 22017478

[111] Tadmor T Shvidel L Goldschmidt N Ruchlemer R Fineman R Bairey O. Hodgkin's Variant of Richter Transformation in Chronic Lymphocytic Leukemia; a Retrospective Study From the Israeli CLL Study Group. Anticancer Res (2014) 34:785–90. 24511013

[112] Martin WG Ristow KM Habermann TM Colgan JP Witzig TE Ansell SM. Bleomycin Pulmonary Toxicity has a Negative Impact on the Outcome of Patients With Hodgkin’s Lymphoma. J Clin Oncol (2005) 23:7614–20. doi: 10.1200/JCO.2005.02.7243 16186594

[113] Johnson P Federico M Kirkwood A Fossa A Berkahn L Carella A. Adapted Treatment Guided by Interim PET-CT Scan in Advanced Hodgkin’s Lymphoma. N Engl J Med (2016) 374:2419–29. doi: 10.1056/NEJMoa1510093 PMC4961236 27332902

[114] El Hussein S Chen P Medeiros LJ Wistuba II Jaffray D Wu J. Artificial Intelligence Strategy Integrating Morphologic and Architectural Biomarkers Provides Robust Diagnostic Accuracy for Disease Progression in Chronic Lymphocytic Leukemia. J Pathol (2021) 256(1):4–14. doi: 10.1002/path.5795 34505705 PMC9526447

[115] Albano D Camoni L Rodella C Giubbini R Bertagna F. 2-[18f]-FDG PET/CT Role in Detecting Richter Transformation of Chronic Lymphocytic Leukemia and Predicting Overall Survival. Clin Lymphoma Myeloma Leuk (2021) 21(3):e277–83. doi: 10.1016/j.clml.2020.12.003 33386279

[116] Pontoizeau C Girard A Mesbah H Haumont LA Devillers A Tempescul A. Prognostic Value of Baseline Total Metabolic Tumor Volume Measured on FDG PET in Patients With Richter Syndrome. Clin Nucl Med (2020) 45:118–22. doi: 10.1097/RLU.0000000000002879 31876819

[117] Musanhu E Sharma RK Attygalle A Wotherspoon A Chau I Cunningham D. Chronic Lymphocytic Leukaemia and Richter’s Transformation: Multimodal Review and New Imaging Paradigms. Clin Radiol (2021) 76:789–800. doi: 10.1016/j.crad.2021.06.001 34217434

[118] Choi MY Widhopf GF II Wu CC Cui B Lao F Sadarangani A. Preclinical Specificity and Safety of UC-961, a First-in-Class Monoclonal Antibody Targeting ROR1. Clin Lymphoma Myeloma Leuk (2015) 15(suppl):S167–9. doi: 10.1016/j.clml.2015.02.010 PMC4548279 26297272

[119] Choi MY Widhopf GF 2nd Ghia EM Kidwell RL Hasan MK Yu J. Phase I Trial: Cirmtuzumab Inhibits ROR1 Signaling and Stemness Signatures in Patients With Chronic Lymphocytic Leukemia. Cell Stem Cell (2018) 22(6):951–959.e3. doi: 10.1016/j.stem.2018.05.018 29859176 PMC7001723

[120] Vaisitti T Arruga F Vitale N Lee TT Ko M Chadburn A. ROR1 Targeting With the Antibody-Drug Conjugate VLS-101 is Effective in Richter Syndrome Patient-Derived Xenograft Mouse Models. Blood (2021) 137(24):3365–77. doi: 10.1182/blood.2020008404 PMC8212514 33512452

[121] Schmid T Maier J Martin M Tasdogan A Tausch E Barth TFE. U-RT1 - A New Model for Richter Transformation. Neoplasia (2021) 23(1):140–8. doi: 10.1016/j.neo.2020.11.010 PMC7736907 33316538

[122] Fiskus W Mill CP Perera D Birdwell C Deng Q Yang H. BET Proteolysis Targeted Chimera-Based Therapy of Novel Models of Richter Transformation-Diffuse Large B-Cell Lymphoma. Leukemia (2021) 35(9):2621–34. doi: 10.1038/s41375-021-01181-w PMC8410602 33654205

[123] Iannello A Vitale N Coma S Arruga F Chadburn A Di Napoli A. Synergistic Efficacy of the Dual PI3K-δ/γ Inhibitor Duvelisib With the Bcl-2 Inhibitor Venetoclax in Richter Syndrome PDX Models. Blood (2021) 137(24):3378–89. doi: 10.1182/blood.2020010187 33786583

[124] Scarfò I Ormhøj M Frigault MJ Castano AP Lorrey S Bouffard AA. Anti-CD37 Chimeric Antigen Receptor T Cells are Active Against B- and T-Cell Lymphomas. Blood (2018) 132(14):1495–506. doi: 10.1182/blood-2018-04-842708 PMC6172564 30089630

[125] Zhang Y Li J Lou X Chen X Yu Z Kang L. A Prospective Investigation of Bispecific CD19/22 CAR T Cell Therapy in Patients With Relapsed or Refractory B Cell Non-Hodgkin Lymphoma. Front Oncol (2021) 11:664421. doi: 10.3389/fonc.2021.664421 34113569 PMC8185372

